# Immeasurable Time Bias in Self-controlled Designs: Case-crossover, Case-time-control, and Case-case-time-control Analyses

**DOI:** 10.2188/jea.JE20210099

**Published:** 2023-02-05

**Authors:** Han Eol Jeong, Hyesung Lee, In-Sun Oh, Kristian B. Filion, Ju-Young Shin

**Affiliations:** 1 School of Pharmacy, Sungkyunkwan University, Suwon, Gyeonggi-do, Republic of Korea; 2 Departments of Medicine and of Epidemiology, Biostatistics, and Occupational Health, McGill University, Montreal, Quebec, Canada; 3 Centre for Clinical Epidemiology, Lady Davis Research Institute - Jewish General Hospital, Montreal, Quebec, Canada; 4 Department of Clinical Research Design & Evaluation, Samsung Advanced Institute for Health Sciences & Technology, Sungkyunkwan University, Seoul, Republic of Korea

**Keywords:** healthcare database, immeasurable time bias, pharmacoepidemiologic study, self-controlled designs

## Abstract

**Background:**

Impact of immeasurable time bias (IMTB) is yet to be examined in self-controlled designs.

**Methods:**

We conducted case-crossover, case-time-control, and case-case-time-control analyses using Korea’s healthcare database. Two empirical examples among elderly patients were used: 1) benzodiazepines-hip fracture; 2) benzodiazepines-mortality. For cases, the date of hip fracture diagnosis or death was defined as the index date, and the inherited date of their matched cases for controls or future cases. Exposure was assessed in the 1–30 day (hazard) and 61–90 day (control) windows preceding the index date. A non-missing exposure setting included in- and outpatient prescriptions and the pseudo-outpatient setting included only the outpatients. Conditional logistic regression was done to estimate odds ratios (ORs) with 95% confidence intervals (CIs), where the relative difference in OR among the two settings was calculated to quantify the IMTB.

**Results:**

The IMTB had negligible impacts in the hip fracture example in the case-crossover (non-missing exposure setting OR 1.27; 95% CI, 1.12–1.44; pseudo-outpatient setting OR 1.21; 95% CI, 1.06–1.39; magnitude 0.05), case-time-control (OR 1.18; 95% CI, 0.98–1.44; OR 1.13; 95% CI, 0.92–1.38; 0.04, respectively), and case-case-time-control analyses (OR 0.99; 95% CI, 0.80–1.23; OR 0.94; 95% CI, 0.75–1.18; 0.05, respectively). In the mortality example, IMTB had significant impacts in the case-crossover (non-missing exposure setting OR 1.44; 95% CI, 1.36–1.52; pseudo-outpatient setting OR 0.72; 95% CI, 0.67–0.78; magnitude 1.00), case-time-control (OR 1.38; 95% CI, 1.26–1.51; OR 0.68; 95% CI, 0.61–0.76; 1.03, respectively), and case-case-time-control analyses (OR 1.27; 95% CI, 1.15–1.40; OR 0.62; 95% CI, 0.55–0.69; 1.05, respectively).

**Conclusion:**

Although IMTB had negligible impacts on the drug’s effect on acute events, as these are unlikely to be accompanied with hospitalizations, it negatively biased the drug’s effect on mortality, an outcome with prodromal phases, in the three self-controlled designs.

## INTRODUCTION

Pharmacoepidemiologic studies using self-controlled designs are becoming more popular owing to their characteristic of inherently controlling for time-invariant confounders. Regardless to the design used, immeasurable time bias becomes evident when the exposure history of persons who become cases is differentially obscured in the time preceding hospitalization, during which dispensing or prescription of medications are not recorded in various administrative healthcare databases.^1^^,^^2^ While a few studies have examined this bias in more traditional designs of cohort^3^ or case-control designs,^4^ which found exaggerated benefit of drugs on mortality, its assessment in self-controlled designs is limited.

Immeasurable time bias is problematic in either hospitalized patients, those at a higher risk of any outcome for which there may be a prodromal phase (eg, death), or patients with chronic disease, with hospitalizations more likely to be frequent and lengthy. When drug data that are dispensed while a patient is hospitalized are missing from the healthcare databases used, these patients could be misclassified as being unexposed to exaggerate a drug’s benefit associated with an outcome.^1^^–^^4^ As long as hospitalizations and the consequent interval-censoring of exposure is predictive of later case status, a negative bias in estimates of drug-exposure effects will be present. Self-controlled designs, developed to investigate the relationship between intermittent exposure and onset of acute events, make within-person comparisons among cases. Thus, immeasurable time bias may be more pronounced in periods preceding the outcome of interest among cases by having frequent and prolonged hospitalizations.^5^^–^^8^ Hence, given the increase in use of these designs and potential threats to those findings’ validity persisting, it is important to better understand the immeasurable time bias in them.^9^^–^^11^

Three self-controlled designs, the case-crossover,^12^ and its two variants, case-time-control^13^ and case-case-time-control,^14^ were of interest, to demonstrate the presence, direction, and magnitude of immeasurable time bias using the nationwide healthcare database of South Korea, which contains in- and outpatient medication information.

## METHODS

### Data source

We used the National Health Insurance Service-National Sample Cohort (NHIS-NSC) of South Korea, described in more detail previously elsewhere.^15^ The NHIS-NSC is a 2.2% randomly extracted database comprising of one million people, with its validity and representativeness confirmed.^15^^,^^16^ The NHIS-NSC contains information on sociodemographic variables (eg, age, sex), healthcare utilization (eg, procedures, hospitalization duration), diagnoses (International Classification of Disease 10^th^ Revision [ICD-10] codes), and prescriptions (eg, national drug codes, day’s supply, dosage) from all settings, encompassing both inpatient, outpatient, and nursing home stays; this is possible as South Korea uses a fee-for-service system for reimbursement. The NHIS-NSC database also provides death-related information (cause of death [ICD-10], date of death), which were linked from South Korea’s national vital statistics.^15^

### Empirical studies

With self-controlled designs designed to assess the relationship between intermittent exposures and acute events, we investigated the immeasurable time bias using two empirical examples that fit this description. Among elderly patients aged ≥65 years, our first example aimed to assess whether transient use of benzodiazepine was a trigger for hip fracture, and our second example aimed to examine the same exposure as a trigger for all-cause death. The range for the expected magnitude of effect for benzodiazepines and hip fracture was from 1.34 (95% confidence interval [CI], 1.26–1.44)^17^ to 1.52 (95% CI, 1.37–1.68),^18^ based on pooled relative risk estimates from two meta-analyses. The corresponding range for benzodiazepines and mortality was from 1.40 (95% CI, 1.30–1.50)^19^ to 1.60 (95% CI, 1.03–2.49),^20^ based on one case-crossover study and a meta-analysis, respectively. We chose elderly patients, as they are more likely to experience health deterioration over time to result in frequent and lengthy hospitalizations. Hence, immeasurable time bias is believed to have considerable impacts in this age population.

### Study subjects

**Figure 1.**
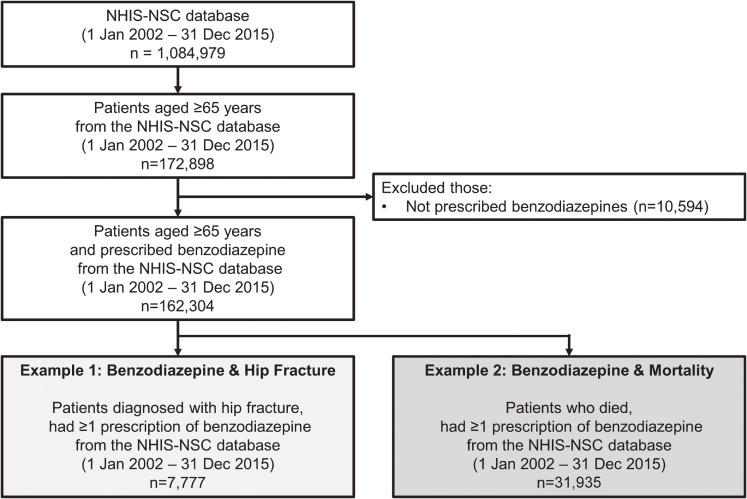
Study subject selection flow chart using Korea’s nationwide healthcare database **Note**: NHIS-NSC, National Health Insurance Service-National Sample Cohort

A cohort of patients aged ≥65 years between 2002 and 2015 were identified (eTable 1). We defined cohort entry as the earliest of: January 1 of the year when a patient turned 65 years or January 1, 2002. Patients not prescribed benzodiazepines after cohort entry were excluded, as they are used for future self-controlled analyses (Figure 1).

### Self-controlled analyses

Immeasurable time bias was assessed in three self-controlled designs of the case-crossover, case-time-control, and case-case-time-control. First, in the case-crossover analysis, exposure to benzodiazepines was compared in two windows within cases (diagnosed with hip fracture [*n* = 7,777]; died [*n* = 31,935]).^12^ The date of diagnosis with hip fracture (ICD-10: S72) from any care setting using primary or secondary diagnosis codes or death was designated as the index date. For the two exposure assessment windows (hazard, control), the hazard window was defined as the 1–30 days and the control window was defined as the 61–90 days preceding the index date. To minimize any autocorrelation and carryover effects in exposure, we introduced a washout window of 30 days.^10^ Using the date of prescription and the days’ supply, we created binary indicators for exposure in the two windows, where a patient was considered exposed when there was ≥1 day of exposed time within the window. For a prescription record of benzodiazepine to be eligible as an exposure, it had to meet any one of the following: (1) benzodiazepine that was prescribed within the window; (2) benzodiazepine that was prescribed prior to the window period but the end date of prescription (days’ supply added to the prescription date) lied within the window; (3) benzodiazepine that was prescribed prior to the window period and the end date of prescription being after the window.

We conducted additional crossover analysis among controls in the case-time-control analysis to control for any temporal trends in exposure, if present.^13^ Controls were all those who did not experience hip fracture or alive at the calendar time of matching. One case was matched to one control using risk-set sampling on sex, age (2-year caliper), time period from cohort entry to the index date, and date of cohort entry (90-day caliper)^21^; this resulted in 7,777 and 31,928 case-control pairs. Finally, we used future cases, or controls at the calendar time of matching (become cases in a later date), in the case-case-time-control analysis as selection bias could occur from inaccurate control sampling.^14^ One future case was matched to each case using the equivalent risk-set sampling^21^ criteria aforementioned, with one added criteria to assure exposure independence: future cases experienced the study outcome in the 90–365 day period after their respective cases’ index date^14^; a total of 7,497 and 30,874 case-future case pairs were identified. The index date for controls and future cases were their respective matched cases’ index dates. Exposure assessment in the case-crossover variant analyses was identical to the case-crossover analysis, to estimate an overall odds ratio (OR) by dividing the case OR over the control (future case) OR.

### Identification of immeasurable time bias

**Figure 2.**
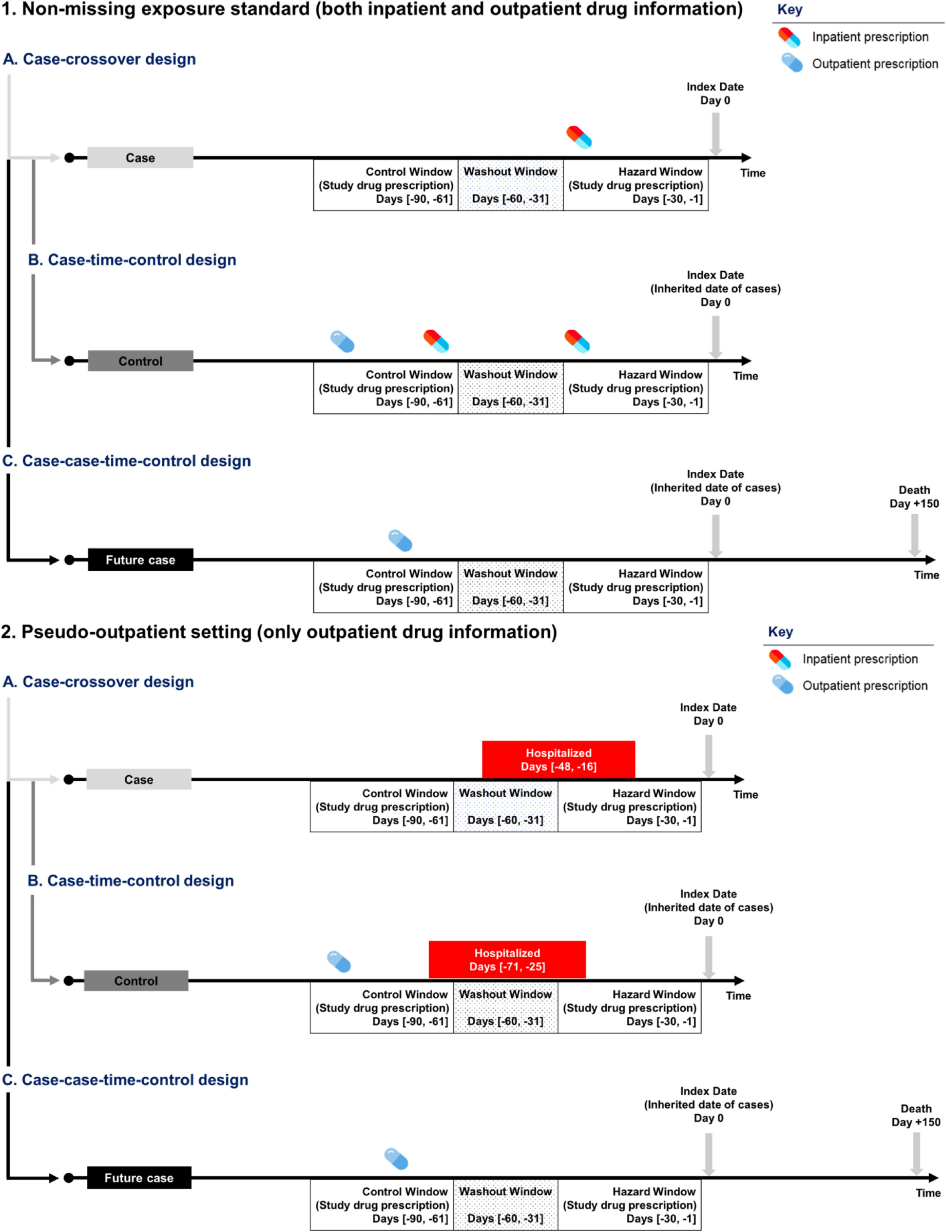
Schematic of the case-crossover, case-time-control and case-case-time-control designs, accompanied by depicting when and how the immeasurable time bias may have occurred in each designs.

Two exposure settings were created to demonstrate immeasurable time bias (Figure 2). A non-missing exposure setting was defined using benzodiazepine prescriptions from all settings and a pseudo-outpatient setting using only outpatient data. The latter setting was created to artificially mask inpatient drug data. To quantify the immeasurable time bias, we estimated the relative difference in ORs obtained from the two settings, where 0 infers no immeasurable time bias. Moreover, three analytical approaches were conducted to mitigate immeasurable time bias: 1) excluded patients hospitalized in either window; 2) adjusted for hospitalization as a dichotomous variable; 3) stratified for the presence of hospitalization.

### Statistical analysis

Conditional logistic regression analysis, conditioned on each patient, was done to estimate ORs and their corresponding 95% CIs for the use of benzodiazepine associated with hip fracture or mortality; only discordant exposure pairs among the two windows were used for analysis. We estimated the OR in the two exposure settings to identify the presence of immeasurable time bias and assess its direction and magnitude. With time-invariant confounders implicitly adjusted for in self-controlled designs, only time-variant confounders were included in the regression models for adjustment (eTable 1). All statistical analyses were conducted using SAS Enterprise Guide 7.1 (SAS Institute Inc., Cary, NC, USA).

## RESULTS

### Empirical study 1: benzodiazepine use and hip fracture

**Figure 3.**
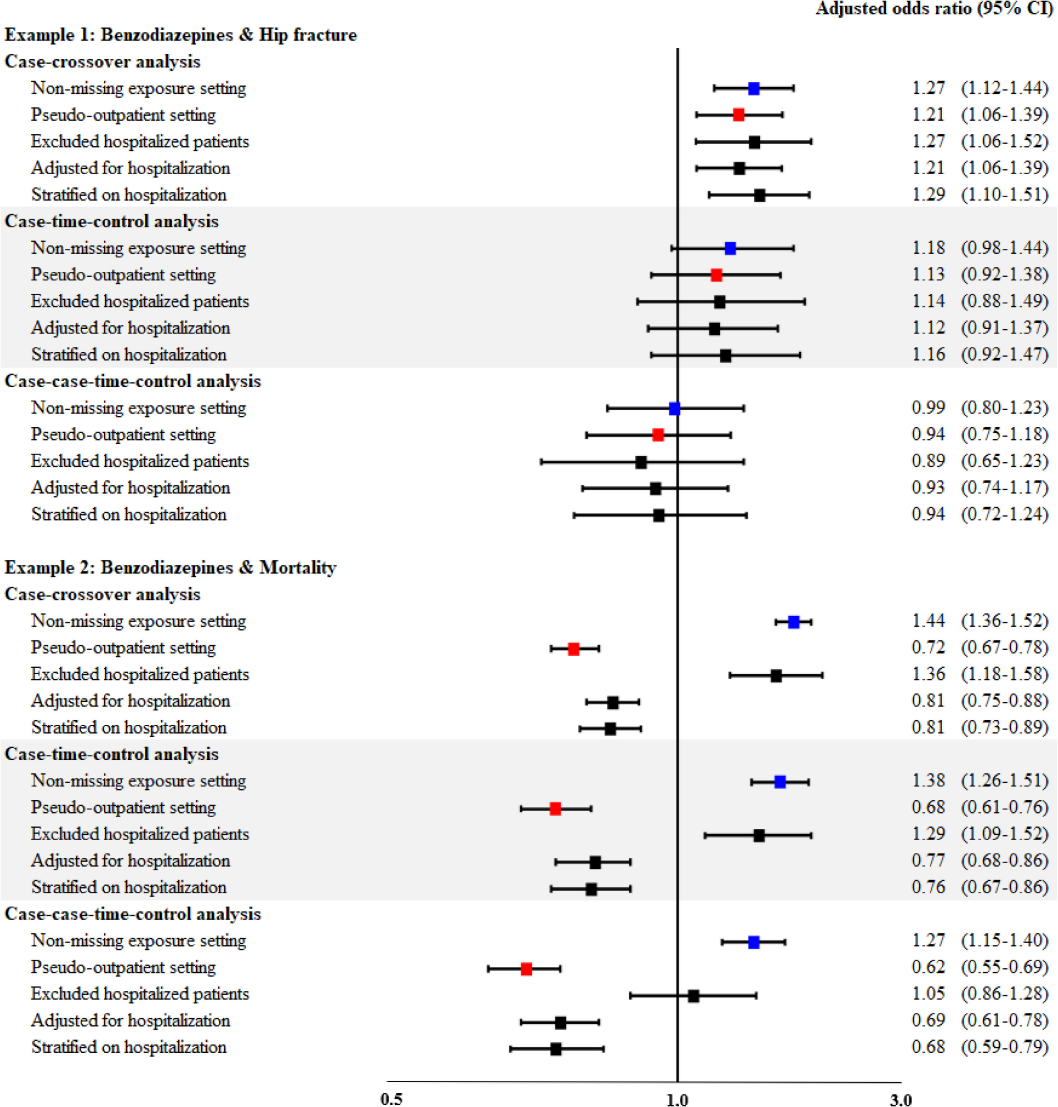
Forest plot summarizing the adjusted odds ratios of the non-missing exposure setting (inpatient and outpatient drug information), pseudo-outpatient setting (only outpatient drug information), and methodological approaches to address immeasurable time bias from two case example studies in three self-controlled designs. ^*^For adjusted odds ratios of the case-time-control and case-case-time-control designs, these were the ratio of the odds ratio of cases and their control and future case, respectively.

Of 162,304 elderly patients, 7,777 cases were identified. Cases were mostly female (72.5%), with a mean age of 70.8 years and had a higher proportion of co-medication use and comorbidities in the hazard window. The case-time-control and case-case-time-control analyses had 7,777 and 7,497 pairs, respectively, where in all self-controlled analyses, cases had a higher proportion of hospitalization in the hazard window; similar proportion were found for controls and future cases (Table 1).[Table tbl02] The number of discordant exposure pairs in the two windows was similar in both exposure settings in the three analyses (Table 3). The association between benzodiazepine use and risk of hip fracture was consistent in both exposure settings of the case-crossover (non-missing exposure setting OR 1.27; 95% CI, 1.12–1.44 vs pseudo-outpatient setting OR 1.21; 95% CI, 1.06–1.39), case-time-control (non-missing exposure setting OR 1.18; 95% CI, 0.98–1.44 vs pseudo-outpatient setting OR 1.13; 95% CI, 0.92–1.38), and case-case-time-control analyses (non-missing exposure setting OR 0.99; 95% CI, 0.80–1.23 vs pseudo-outpatient setting OR 0.94; 95% CI, 0.75–1.18) (Figure 3). In support, the immeasurable time bias was negligible in the three self-controlled analyses with a magnitude of 0.05 (case-crossover), 0.04 (case-time-control), and 0.05 (case-case-time-control) (Table 3).

**Table 1.** Baseline characteristics of subjects from the empirical example of benzodiazepine exposure as a potential trigger for hip fracture among elderly patients aged ≥65 years

Characteristics	Case-crossover analysis				Case-time-control analysis (1:1 matching)								Case-case-time-control analysis (1:1 matching)							
		
	Cases (*n* = 7,777)				Cases (*n* = 7,777)				Controls (*n* = 7,777)				Cases (*n* = 7,497)				Future cases^a^ (*n* = 7,497)			
**Age,^b^ years, mean (SD)**	70.8 (5.7)				70.8 (5.7)				70.9 (5.7)				71.0 (5.7)				71.0 (5.7)			
**Male^b^**	2,136		27.5		2,136		27.5		2,136		27.5		2,039		27.2		2,039		27.2	
	**Hazard**		**Control**		**Hazard**		**Control**		**Hazard**		**Control**		**Hazard**		**Control**		**Hazard**		**Control**	
**Hospitalization^c^**	967	34.8	790	28.4	967	34.8	790	28.4	461	23.1	461	23.1	948	35.2	777	28.8	616	46.6	581	44.0
**Comorbidities^c^**																				
Asthma	124	4.5	118	4.2	124	4.5	118	4.2	57	2.9	63	3.2	119	4.4	115	4.3	112	8.5	91	6.9
Atrial fibrillation	23	0.8	20	0.7	23	0.8	20	0.7	10	0.5	14	0.7	23	0.9	19	0.7	20	1.5	16	1.2
COPD	147	5.3	143	5.1	147	5.3	143	5.1	88	4.4	79	4.0	145	5.4	140	5.2	143	10.8	135	10.2
Ischemic heart disease	146	5.3	121	4.4	146	5.3	121	4.4	91	4.6	83	4.2	143	5.3	116	4.3	101	7.6	91	6.9
Diabetes mellitus	432	15.6	419	15.1	432	15.6	419	15.1	255	12.8	252	12.7	419	15.6	406	15.1	304	23.0	290	22.0
Hyperlipidemia	138	5.0	136	4.9	138	5.0	136	4.9	119	6.0	112	5.6	132	4.9	130	4.8	109	8.3	106	8.0
Hypertension	901	32.4	929	33.4	901	32.4	929	33.4	746	37.4	743	37.3	872	32.4	898	33.3	679	51.4	671	50.8
Renal failure	54	1.9	45	1.6	54	1.9	45	1.6	11	0.6	6	0.3	52	1.9	43	1.6	30	2.3	31	2.3
Stroke	174	6.3	180	6.5	174	6.3	180	6.5	73	3.7	69	3.5	169	6.3	173	6.4	130	9.8	121	9.2
Myocardial infarction	6	0.2	12	0.4	6	0.2	12	0.4	10	0.5	6	0.3	6	0.2	11	0.4	12	0.9	8	0.6
Heart failure	170	6.1	174	6.3	50	1.8	42	1.5	23	1.2	33	1.7	160	5.9	164	6.1	104	7.9	103	7.8
Malignancy	78	2.8	68	2.4	78	2.8	68	2.4	49	2.5	44	2.2	78	2.9	66	2.4	61	4.6	50	3.8
Rheumatoid arthritis	44	1.6	49	1.8	44	1.6	49	1.8	17	0.9	25	1.3	43	1.6	47	1.7	33	2.5	40	3.0
Osteoarthritis	491	17.7	472	17.0	491	17.7	472	17.0	304	15.3	314	15.8	473	17.6	452	16.8	371	28.1	398	30.1
**Co-medications^c^**																				
ACE inhibitors	156	5.6	131	4.7	156	5.6	131	4.7	95	4.8	64	3.2	153	5.7	128	4.8	106	8.0	96	7.3
ARBs	716	25.8	579	20.8	716	25.8	579	20.8	488	24.5	390	19.6	690	25.6	555	20.6	469	35.5	412	31.2
β-blockers	529	19.0	435	15.7	529	19.0	435	15.7	385	19.3	309	15.5	512	19.0	422	15.7	357	27.0	306	23.2
Antidiabetic drugs	561	20.2	474	17.1	561	20.2	474	17.1	315	15.8	268	13.5	545	20.2	458	17.0	355	26.9	319	24.1
CCBs	985	35.5	815	29.3	985	35.5	815	29.3	686	34.4	583	29.3	957	35.5	796	29.5	690	52.2	615	46.6
Statins	501	18.0	383	13.8	501	18.0	383	13.8	356	17.9	268	13.5	479	17.8	363	13.5	327	24.8	276	20.9
Opioids	1,020	36.7	892	32.1	1,020	36.7	892	32.1	542	27.2	559	28.1	985	36.6	860	31.9	656	49.7	635	48.1
Antidepressants	645	23.2	537	19.3	645	23.2	537	19.3	283	14.2	263	13.2	620	23.0	511	19.0	381	28.8	337	25.5
NSAIDs	1,548	55.7	1,356	48.8	1,548	55.7	1,356	48.8	960	48.2	950	47.7	1,496	55.5	1,311	48.7	1,002	75.9	958	72.5
Acetaminophen	992	35.7	894	32.2	992	35.7	894	32.2	579	29.1	555	27.9	958	35.6	862	32.0	689	52.2	669	50.6

ACE, angiotensin-converting enzyme; ARB, angiotensin receptor blockers; CCB, calcium channel blockers; COPD, chronic obstructive pulmonary disease; NA, not applicable; NSAIDs, nonsteroidal anti-inflammatory drugs; SD, standard deviation.

Values are numbers (percentages), unless otherwise stated.

^a^Cases that had not yet experienced study outcome at the point in time when matching was done, but eventually become cases over time.

^b^Measured at the index date.

^c^Measured in the hazard (1–30 days before the index date) and control (61 to 90 days before the index date) windows.

### Empirical study 2: benzodiazepine use and mortality

Of 162,304 elderly patients, 31,935 cases were identified, where cases were mostly female (51.2%), with a mean age of 79.3 years; clinical characteristics were similar to that of empirical study 1. There were 31,928 case-control and 30,874 case-future case pairs identified (Table 2). The number of discordant exposure pairs in the two windows were distinctively reduced in the pseudo-outpatient setting when compared to the non-missing exposure setting in all analyses; proportion of exposed became similar in the two windows in the pseudo-outpatient setting. However, this proportion remained consistent among controls or future cases (Table 3). The non-missing exposure setting case-crossover (OR 1.44; 95% CI, 1.36–1.52), case-time-control (OR 1.38; 95% CI, 1.26–1.51), and case-case-time-control analyses (OR 1.27; 95% CI, 1.15–1.40) showed that benzodiazepine use was associated with an increased risk of mortality. Meanwhile, a protective association was found from the pseudo-outpatient setting from all three analyses, suggesting that the immeasurable time bias had significant impacts (case-crossover: OR 0.72; 95% CI, 0.67–0.78; case-time-control: OR 0.68; 95% CI, 0.61–0.76; case-case-time-control: OR 0.62; 95% CI, 0.55–0.69) (Figure 3). The variation in the immeasurable time bias’s magnitude was minimal in the three self-controlled designs: 1.00 (case-crossover), 1.03 (case-time-control), and 1.05 (case-case-time-control) (Table 3).

**Table 2. tbl02:** Baseline characteristics of subjects from the empirical example of benzodiazepine exposure as a potential trigger for all-cause mortality among elderly patients aged ≥65 years

Characteristics	Case-crossover analysis				Case-time-control analysis (1:1 matching)								Case-case-time-control analysis (1:1 matching)							
		
	Cases (*n* = 31,935)				Cases (*n* = 31,928)				Controls (*n* = 31,928)				Cases (*n* = 30,874)				Future cases^a^ (*n* = 30,874)			
**Age,^b^ years, mean (SD)**	79.3 (7.4)				79.3 (7.4)				79.0 (7.2)				79.2 (7.4)				79.2 (7.3)			
**Male^b^**	15,575		48.8		15,573		48.8		15,573		48.8		15,060		48.8		15,064		48.8	
	**Hazard**		**Control**		**Hazard**		**Control**		**Hazard**		**Control**		**Hazard**		**Control**		**Hazard**		**Control**	
**Hospitalization^c^**	11,708	78.6	6,828	45.8	11,707	78.5	6,828	45.8	914	12.0	916	12.0	11,384	78.6	6,652	45.9	3,185	44.7	2,673	37.5
**Comorbidities^c^**																				
Asthma	716	4.8	741	5.0	715	4.8	741	5.0	250	3.3	244	3.2	700	4.8	728	5.0	497	7.0	474	6.7
Atrial fibrillation	238	1.6	231	1.5	238	1.6	231	1.5	66	0.9	62	0.8	229	1.6	224	1.5	135	1.9	113	1.6
COPD	1,229	8.2	1,278	8.6	1,229	8.2	1,278	8.6	384	5.0	368	4.8	1,197	8.3	1,248	8.6	769	10.8	729	10.2
Ischemic heart disease	682	4.6	724	4.9	682	4.6	724	4.9	365	4.8	372	4.9	663	4.6	707	4.9	490	6.9	477	6.7
Diabetes mellitus	1,652	11.1	2,139	14.4	1,651	11.1	2,138	14.3	977	12.8	956	12.5	1,590	11.0	2,062	14.2	1,301	18.3	1,299	18.2
Hyperlipidemia	279	1.9	480	3.2	279	1.9	479	3.2	503	6.6	482	6.3	269	1.9	458	3.2	309	4.3	339	4.8
Hypertension	2,883	19.3	4,011	26.9	2,883	19.3	4,011	26.9	2,921	38.2	2,928	38.3	2,797	19.3	3,899	26.9	2,533	35.6	2,607	36.6
Renal failure	826	5.5	589	4.0	826	5.5	589	4.0	55	0.7	49	0.6	795	5.5	568	3.9	287	4.0	270	3.8
Stroke	1,392	9.3	1,093	7.3	1,392	9.3	1,093	7.3	267	3.5	256	3.4	1,358	9.4	1,064	7.3	677	9.5	631	8.9
Myocardial infarction	335	2.2	123	0.8	335	2.2	123	0.8	28	0.4	19	0.2	328	2.3	121	0.8	65	0.9	43	0.6
Heart failure	1,353	9.1	1,354	9.1	1,353	9.1	1,354	9.1	341	4.5	333	4.4	1,312	9.1	1,312	9.1	606	8.5	602	8.5
Malignancy	4,277	28.7	3,372	22.6	4,277	28.7	3,372	22.6	186	2.4	149	2.0	4,149	28.6	3,268	22.6	1,304	18.3	1,051	14.8
Rheumatoid arthritis	94	0.6	145	1.0	94	0.6	145	1.0	42	0.5	52	0.7	93	0.6	143	1.0	71	1.0	74	1.0
Osteoarthritis	664	4.5	1,130	7.6	664	4.5	1,130	7.6	1,126	14.7	148	1.9	644	4.4	1,096	7.6	917	12.9	892	12.5
**Co-medications^c^**																				
ACE inhibitors	1,029	6.9	785	5.3	1,029	6.9	785	5.3	305	4.0	257	3.4	1,007	7.0	776	5.4	503	7.1	425	6.0
ARBs	3,468	23.3	3,118	20.9	3,467	23.3	3,117	20.9	2,264	29.6	1,806	23.6	3,374	23.3	3,037	21.0	2,088	29.3	1,799	25.3
β-blockers	3,162	21.2	2,398	16.1	3,162	21.2	2,398	16.1	1,336	17.5	1,075	14.1	3,090	21.3	2,348	16.2	1,544	21.7	1,333	18.7
Antidiabetic drugs	4,075	27.3	2,952	19.8	4,074	27.3	2,951	19.8	1,165	15.2	983	12.9	3,967	27.4	2,870	19.8	1,665	23.4	1,494	21.0
CCBs	4,734	31.8	4,070	27.3	4,733	31.8	4,069	27.3	2,729	35.7	2,283	29.9	4,609	31.8	3,964	27.4	2,550	35.8	2,274	31.9
Statins	2,104	14.1	1,697	11.4	2,103	14.1	1,696	11.4	1,584	20.7	1,152	15.1	2,021	13.9	1,628	11.2	1,184	16.6	981	13.8
Opioids	7,622	51.1	5,702	38.3	7,622	51.1	5,702	38.3	2,131	27.9	2,094	27.4	7,399	51.1	5,526	38.1	2,874	40.4	2,601	36.5
Antidepressants	2,495	16.7	2,194	14.7	2,495	16.7	2,194	14.7	1,119	14.6	939	12.3	2,420	16.7	2,121	14.6	1,536	21.6	1,333	18.7
NSAIDs	6,966	46.7	6,108	41.0	6,965	46.7	6,108	41.0	3,590	47.0	3,375	44.2	6,807	47.0	5,954	41.1	3,622	50.9	3,479	48.9

ACE, angiotensin-converting enzyme; ARB, angiotensin receptor blockers; CCB, calcium channel blockers; COPD, chronic obstructive pulmonary disease; NA, not applicable; NSAIDs, nonsteroidal anti-inflammatory drugs; SD, standard deviation.

Value are numbers (percentages), unless otherwise stated.

^a^Cases that had not yet experienced study outcome at the point in time when matching was done, but eventually become cases over time.

^b^Measured at the index date.

^c^Measured in the hazard (1–30 days before the index date) and control (61 to 90 days before the index date) windows.

**Table 3.** Adjusted odds ratios of the non-missing exposure setting (inpatient and outpatient drug information) and pseudo-outpatient setting (only outpatient drug information) for the two case example studies in three self-controlled designs

	Case-crossover		Case-time-control		Case-case-time-control	
		
	Non-missing Exposure	Pseudo-Outpatient	Non-missing Exposure	Pseudo-Outpatient	Non-missing Exposure	Pseudo-Outpatient
	
**Example 1: Benzodiazepine & Hip Fracture**	**Cases**					

Case patients, *n*	7,777		7,777		7,497	
Exposed only in the hazard window, *n* (%)	2,035 (73.3)	1,579 (71.8)	2,035 (73.3)	1,579 (71.8)	1,976 (73.3)	1,535 (71.9)
Exposed only in the control window, *n* (%)	1,753 (63.1)	1,384 (62.9)	1,753 (63.1)	1,384 (62.9)	1,703 (63.2)	1,348 (63.1)
Case-crossover OR^a^ (95% CI)	1.27 (1.12–1.44)	1.21 (1.06–1.39)	1.27 (1.12–1.44)	1.21 (1.06–1.39)	1.26 (1.11–1.43)	1.27 (1.07–1.50)
	**Controls**					
Control patients, *n*	NA		7,777		7,497	
Exposed only in the hazard window	NA		1,334 (67.0)	1,155 (66.5)	1,321 (76.8)	1,068 (74.7)
Exposed only in the control window	NA		1,241 (62.3)	1,070 (61.6)	1,205 (70.0)	981 (68.6)
Control-crossover OR^a^ (95% CI)	NA		1.07 (0.92–1.24)	1.08 (0.93–1.26)	1.20 (1.04–1.37)	1.27 (1.06–1.53)
	**Association between benzodiazepines and hip fracture**					
Adjusted OR^b^ (95% CI)	1.27 (1.12–1.44)	1.21 (1.06–1.39)	1.18 (0.98–1.44)	1.13 (0.92–1.38)	0.99 (0.80–1.23)	0.94 (0.75–1.18)
	**Magnitude of immeasurable time bias**					
$\frac{\text{Nonmissing Exposure Setting OR}}{\text{Pseudo Outpatient Setting OR}}-1$	$\frac{1.27}{1.21}-1=0.05$		$\frac{1.18}{1.13}-1=0.04$		$\frac{0.99}{0.94}-1=0.05$	

	Non-missing Exposure	Pseudo-Outpatient	Non-missing Exposure	Pseudo-Outpatient	Non-missing Exposure	Pseudo-Outpatient

**Example 2: Benzodiazepine & Mortality**	**Cases**					
Case patients, *n*	31,935		31,928		30,874	
Exposed only in the hazard window, *n* (%)	10,076 (67.6)	4,036 (60.2)	10,075 (67.6)	4,036 (60.2)	9,787 (67.6)	3,920 (60.1)
Exposed only in the control window, *n* (%)	7,680 (51.5)	4,461 (66.6)	7,680 (51.5)	4,461 (66.6)	7,460 (51.5)	4,339 (66.5)
Case-crossover OR^a^ (95% CI)	1.44 (1.36–1.52)	0.72 (0.67–0.78)	1.44 (1.36–1.52)	0.72 (0.67–0.78)	1.43 (1.36–1.51)	0.72 (0.67–0.78)
	**Controls**					
Control patients, *n*	NA		31,929		30,863	
Exposed only in the hazard window	NA		5,188 (67.9)	4,482 (67.3)	5,193 (72.9)	3,675 (73.4)
Exposed only in the control window	NA		4,866 (63.7)	4,202 (63.1)	4,749 (66.7)	3,412 (68.2)
Control-crossover OR^a^ (95% CI)	NA		1.04 (0.97–1.12)	1.06 (0.98–1.15)	1.13 (1.04–1.22)	1.17 (1.07–1.28)
	**Association between benzodiazepines and all-cause mortality**					
Adjusted OR^b^ (95% CI)	1.44 (1.36–1.52)	0.72 (0.67–0.78)	1.38 (1.26–1.51)	0.68 (0.61–0.76)	1.27 (1.15–1.40)	0.62 (0.55–0.69)
	**Magnitude of immeasurable time bias**					
$\frac{\text{Nonmissing Exposure Setting OR}}{\text{Pseudo Outpatient Setting OR}}-1$	$\frac{1.44}{0.72}-1=1.00$		$\frac{1.38}{0.68}-1=1.03$		$\frac{1.27}{0.62}-1=1.05$	

CI, confidence interval; NA, not applicable; OR, odds ratio.

^a^Adjusted for all time-varying comorbidities and co-medications.

^b^Ratio of odds ratio, case-crossover OR divided by the control-crossover OR.

## DISCUSSION

To our knowledge, this is the first study to have investigated immeasurable time bias in pharmacoepidemiologic research that adopted self-controlled designs. In using South Korea’s healthcare database that provided drug data from all settings (inpatient, outpatient), we successfully identified the immeasurable time bias by presenting its presence, direction, and magnitude in the three self-controlled designs. While the immeasurable time bias consistently underestimated the non-missing exposure setting’s OR in varying magnitudes, it had insignificant impacts in the case study that examined the association between transient drug use and acute event (example 1 in our study). However, in our case study of intermittent drug use and mortality (example 2), immeasurable time bias had significant impacts in all three analyses, regardless to the self-controlled study design used. While we focused on immeasurable time specifically arising from periods of hospitalizations, further studies are needed that investigate into other reasons where immeasurable time may arise, for instance, missing specialist prescriptions when using primary care database (eg, Clinical Practice Research Datalink [CPRD]) or when sicker people drop out of the workforce and thus, become invisible to worker employment records. With CPRD as an example, although prescriptions made during hospital stay will be affected by immeasurable time bias, prescriptions made at discharge with subsequent fills in primary care are likely to be affected in studies of chronic exposures, as only the first prescription will be missed.

With no previous study available that assessed the immeasurable time bias in self-controlled designs, we chose two empirical studies, one that involved an acute event (hip fracture) and another being not acute but significantly important (all-cause mortality). In our first empirical study of benzodiazepine use and hip fracture, the ORs between the two exposure settings had minimal discrepancies. Thus, it may be deduced that immeasurable time bias does not affect the validity of findings when the outcome of interest is acute. Therefore, as expected, approaches of excluding hospitalized patients, and adjusting or stratifying on hospitalization had minor effects. This may be because cases that experience acute events, hip fracture in our example, are unlikely to have experience hospitalizations prior to such event. If there were no or minimal periods of hospitalizations throughout the length of the exposure ascertainment window (90 days preceding the index date in our study), then none or minimal inpatient dispensing or prescriptions would have occurred accordingly. Thus, immeasurable time bias would not arise, as there were practically no inpatient medication records that were unavailable for exposure assessment because, unlike death, the first hospitalization for hip fracture is less likely to be preceded by multiple hospitalizations.

Unlike our first example, the immeasurable time bias had very distinct and significant impacts in all three self-controlled designs assessed in our second empirical study of transient benzodiazepine use and mortality. While the ORs from the non-missing exposure setting showed an elevated risk of mortality associated with benzodiazepines, a contrasting protective association was observed from the pseudo-outpatient setting. This clear discrepancy in the impact of immeasurable time bias between the two example studies may be owed to more cases being hospitalized frequently and for lengthy durations prior to death in the second example study. This would naturally lead to a greater number of prescription records that were made during hospitalizations to be unavailable for assessment, which is supported by the disproportional number of hospitalizations observed in the two windows among cases (Table 1 and Table 2). Moreover, while adjusting or stratifying for hospitalization in each window only marginally reduced immeasurable time bias, excluding hospitalized patients showed some promise. However, such exclusion resulted in wide CIs due to reduced statistical power (approximately 70% of patients were excluded) and simultaneously introduced selection bias in its place. Hence, further investigations are needed to minimize or even possibly overcome the immeasurable time bias when conducting self-controlled designs that set their outcome of interest as mortality or events that are likely to be subsequent of multiple hospitalizations.

When comparing the estimates from the non-missing exposure setting to the expected estimates, it was difficult to make a direct comparison for our example study of benzodiazepines and hip fracture. Our non-missing exposure setting’s estimates (case-crossover OR 1.27; 95% CI, 1.12–1.44; case-time-control OR 1.18; 95% CI, 0.98–1.44; case-case-time-control OR 0.99; 95% CI, 0.80–1.23) for the risk of hip fracture associated with benzodiazepine use were smaller than our expected effect estimates (OR 1.34; 95% CI, 1.26–1.44^17^; OR 1.52; 95% CI, 1.37–1.68).^18^ This difference may be attributed to the fact that the expected effect estimates from these meta-analyses were absent of findings from self-controlled studies. Despite having limitations in making a direct comparison, transient use of benzodiazepines is not likely to trigger the onset of hip fracture while benzodiazepine users, as compared with non-users, may be associated with an increased risk. On the other hand, our findings for benzodiazepine use and mortality (case-crossover OR 1.44; 95% CI, 1.36–1.52; case-time-control OR 1.38; 95% CI, 1.26–1.51; case-case-time-control OR 1.27; 95% CI, 1.15–1.40) were similar to the expected estimates (OR 1.40; 95% CI, 1.30–1.50^19^; OR 1.60; 95% CI, 1.03–2.49),^20^ where positive associations were consistently found from these studies. Our findings were particularly consistent when compared to a case-crossover study that reported a 40% increased risk of mortality associated with benzodiazepine, while our case-crossover study found a 44% increased risk.

A previous study noted that time-varying prognosis in self-controlled designs may result in immeasurable time bias.^22^ As frail patients are likely to stop preventive treatments, such as statins, with their likelihood of hospitalization increasing over time (sick-stopper effect), they are therefore more likely to have immeasurable time.^23^^,^^24^ To mitigate bias arising from the aforementioned, one study proposed the estimation of crossover exposure ORs for referent treatments among cases. This is somewhat similar to the case-time-control analyses, where instead of conducting crossover analysis among a pool of controls, they proposed crossover analysis of referent treatments. However, selection of an inappropriate referent treatment could multiplicatively increase the degree of bias or incompletely adjust for it.^22^ This limitation would also apply to the case-time-control analysis, where selection of an inappropriate sample of controls could introduce selection bias.^13^ Despite all, if inpatient medication records were fully unavailable for exposure assessment in the foreign healthcare databases used by previous studies, they would have been limited in demonstrating the true impact of the immeasurable time bias.

Our study has several strengths. First, by using the NHIS-NSC database of South Korea, which has both representativeness of the entire population and healthcare utilization information from all settings, we clearly demonstrated the true impact of immeasurable time bias.^15^ Second, as benzodiazepines are not available as over-the-counter medications, our findings are subject to minimal exposure misclassification, if any, as we used in- and outpatient records to ascertain exposure. Third, outcome misclassification of diagnosis records and cause of death codes are unlikely to have affected our findings, as a prior validation study reported an overall positive predictive value of 82%^25^ and 92%,^15^ respectively, when comparing electronic health records to claims data. Last, we investigated two drug-outcome relationships of benzodiazepine and hip fracture and benzodiazepine and mortality, to investigate immeasurable time bias. In doing so, we believe that extending our findings to other pairs or clinical situations may be generalizable, under normal assumptions.

However, this study also has few limitations. First, as our cohort was comprised of only Koreans, we were limited in reflecting any genetic or ethnic discrepancies present. However, as immeasurable time bias is a problem associated with missing inpatient prescription records in administrative healthcare databases, we believe this limitation to have had minimal effects to the validity of our findings. Second, the impact immeasurable time bias may in the self-controlled case series design is needed. Third, despite being more advanced designs, the case-time-control and case-case-time-control analyses could not mitigate the immeasurable time bias present in the case-crossover design. We believe this to have been the case, as immeasurable time bias is likely to be a stand-alone bias that is independent from other biases (eg, exposure time trends, selection bias). Fourth, in the second example of benzodiazepines and mortality, the observed elevated risk of death associated with benzodiazepine use may be attributed to factors other than our exposure of interest, despite adjusting for various time-varying comorbidities and co-medication use or biases unconsidered for, such as reverse causality. Fifth, there may have been potential misclassification in the timing of exposure if the dispensed/prescribed medications were taken on an as-needed basis rather than on a fixed daily schedule. Despite being unable to differentiate this due to inherent limitations of healthcare data used in this study, we believe there to have been mixed prescriptions of these types, and thus, non-differential between the hazard and controls windows. This would have had minimal impacts in our effect estimates. In the meantime, a simulation study with an investigator designated effect size would be ideal in exploring and if possible, suggesting approaches to mitigate the immeasurable time bias. Sixth, future studies are warranted to investigate the impact of immeasurable time bias in self-controlled designs that assess chronic exposures, as there is an increasing trend in the use of these designs for such exposures. Last, the external validity of the observed negative association of benzodiazepines and mortality from the pseudo-outpatient setting is limited, as we were unable to identify literature that used foreign datasets for comparison.

Immeasurable time bias was present in self-controlled designs, but only when the outcome of interest was mortality. The bias consistently underestimated the drug’s effect associated with an outcome, whether it was an acute event like hip fracture or an outcome like mortality that has a prodromal phase. Further investigations are needed to develop methodological approaches that could mitigate or overcome the immeasurable time bias when conducting a self-controlled design to assess the effect of a drug on mortality.

## SUPPLEMENTARY MATERIAL

eTable 1. List of drugs and diagnoses included in the study
