# Supplementary material for: Immeasurable Time Bias in Self-controlled Designs: Case-crossover, Case-time-control, and Case-case-time-control Analyses
Source: J Epidemiol. 2023 Feb 5;33(2):82–90. doi: 10.2188/jea.JE20210099 (PMC9794445; doi:10.2188/jea.JE20210099)
Supplement: Supplementary file 1 [file je-33-082-s001.pdf]

eTable 1. List of drugs and diagnoses included in the study

**Study drug (ATC code)**	Benzodiazepine[a] (N05BA, N05CD, N05CE, N05CF)
**Comorbidities (ICD-10)**[b]	Asthma (J45, J46)<br>Atrial fibrillation (I48)<br>Chronic obstructive pulmonary disease (J40-J44, J47)<br>Ischemic heart disease (I20-I25)<br>Diabetes mellitus (E10-E14)<br>Hyperlipidemia (E78)<br>Hypertension (I10-I15)<br>Renal failure (N17-N19)<br>Stroke (I60-I64)<br>Myocardial infraction (I21)<br>Heart failure (I50, I090)<br>Malignancy (C00-C99)<br>Rheumatoid arthritis (M05, M06)<br>Osteoarthritis (M15-M19)
**Co-medications (ATC code)**[c]	Angiotensin-converting enzyme inhibitors (C09AA)<br>Angiotensin receptor II blockers (C09CA)<br>β-blockers (C07)<br>Antidiabetic medications (A10)<br>Calcium channel blockers (C08)<br>Statins (C01AA)<br>Opioids (N02A)<br>Antidepressants (N06A)<br>Nonsteroidal anti-inflammatory drugs (M01A)<br>Acetaminophen (N02BE01)

ATC, Anatomical Therapeutic Chemical; ICD, International Classification of Diseases-10th Revision.

[a] Benzodiazepines included were alprazolam, bromazepam, brotizolam, chlordiazepoxide, clobazam, clonazepam, clorazepate, clotiazepam, diazepam, estazolam, ethyl loflazepate, etizolam, fludiazepam, flurazepam, flunitrazepam, lorazepam, midazolam, nordazepam, oxazepam, pinazepam, tofisopam, triazolam

[b] Acute and chronic disease conditions reported to influence the risk of mortality

[c] Drugs reported to have drug-drug interactions, either pharmacodynamics or pharmacokinetic, with the study drugs
